# Bioprocess data mining using regularized regression and random forests

**DOI:** 10.1186/1752-0509-7-S1-S5

**Published:** 2013-08-12

**Authors:** Syeda Sakira Hassan, Muhammad Farhan, Rahul Mangayil, Heikki Huttunen, Tommi Aho

**Affiliations:** 1 Department of Signal Processing, Tampere University of Technology, Tampere, P.O. Box 553, 33101, Finland; 2 Department of Chemistry and Bioengineering, Tampere University of Technology, Tampere, P.O. Box 541, 33101, Finland

## Abstract

**Background:**

In bioprocess development, the needs of data analysis include (1) getting overview to existing data sets, (2) identifying primary control parameters, (3) determining a useful control direction, and (4) planning future experiments. In particular, the integration of multiple data sets causes that these needs cannot be properly addressed by regression models that assume linear input-output relationship or unimodality of the response function. Regularized regression and random forests, on the other hand, have several properties that may appear important in this context. They are capable, e.g., in handling small number of samples with respect to the number of variables, feature selection, and the visualization of response surfaces in order to present the prediction results in an illustrative way.

**Results:**

In this work, the applicability of regularized regression (Lasso) and random forests (RF) in bioprocess data mining was examined, and their performance was benchmarked against multiple linear regression. As an example, we used data from a culture media optimization study for microbial hydrogen production. All the three methods were capable in providing a significant model when the five variables of the culture media optimization were linearly included in modeling. However, multiple linear regression failed when also the multiplications and squares of the variables were included in modeling. In this case, the modeling was still successful with Lasso (correlation between the observed and predicted yield was 0.69) and RF (0.91).

**Conclusion:**

We found that both regularized regression and random forests were able to produce feasible models, and the latter was efficient in capturing the non-linearity in the data. In this kind of a data mining task of bioprocess data, both methods outperform multiple linear regression.

## Background

Industrial biotechnology exploits processes that use living cells, for instance yeast and various bacteria, to produce products like fine chemicals, active pharmaceutical ingredients, enzymes, and biofuels. The use of living material in manufacturing processes makes the processes challenging to develop and control. Because of the complexity of these tasks, computational modeling and data analysis are used to improve the yield, reproducibility and robustness in bioprocesses. On the other hand, the regulatory demands on pharmaceutical manufacturing processes are increasing and, for example, the United States Food and Drug Administration emphasize the importance of model-aided process development in its process analytical technology (PAT) initiative [1]. One of the important steps in process development is maximizing the product yield. In practice, the process optimization includes (1) identifying the process parameters that have most impact to the product yield and, (2) determining their optimal values. This data analysis task includes few features that are specific to the application area. For example, the number of process parameters (predictors) may be large with respect to the number of samples, the predictors may contain either numerical or categorical values, the datasets may contain missing values and, finally, the relationship among the predictors and product yield may be non-linear.

To build a model for data analysis requires selection of important features while leaving out the rest. Several feature selection methods have been proposed but the results tend to vary, as generalization of the solution is problematic. Typical issues are data redundancy, outliers and feature dependencies [2,3].

## Methods

In this work, we have used three alternative approaches to model bioprocess data: multiple linear regression, regularized regression and random forests. The analyses were performed using MATLAB [4] and RF-ACE tool [5].

### Multiple linear regression

In multiple linear regression, the response variable is modeled as a linear combination of multiple predictor variables. The general model can be expressed as

(1) $$y=\beta_{0}+a_{1}\beta_{1}+a_{2}\beta_{2}+a_{3}\beta_{3}+\ldots +a_{p}\beta_{p}$$

where *y *is the response variable, and *a_i _*and *β_i _*(*i = *1*, ..., p*) are the predictor variables and their coefficients, respectively. The intercept is represented by *β_0_*. Alternatively, Equation (1) can be represented in vector notation by **y = H***θ*, where **H **is augmented predictor vector given as [**1 *a*_1 _*a*_2 _*... a_p_***] and *θ *is the parameter vector.

In spite of being linear with respect to the predictor variables, multiple linear regression models fail to incorporate the underlying non-linear relationships, if it exists, between the predictors and the response variable. However, the model restricts only the coefficients to be linearly related, while the predictor variables can be non-linear. This gives a provision of including additional non-linearly transformed predictor variables in the linear regression modeling. The advantage of using such variables in regression analysis is that the non-linear behavior in data and interaction between different variables are incorporated while the model remains linear and easily interpretable. This is a typical procedure applied in traditional response surface modeling when constructing models with quadratic terms and interactions of terms. Increasing the number of parameters in this way, however, causes high-dimensional predictor vector which results in over-fitting and the loss of generality. Moreover, if the number of samples is small, increasing the parameter vector size by these transformations may cause rank deficiency or multicollinearity of the prediction vector. In such cases, standard regression modeling may either fail, rank deficiency may cause non-invertible matrix thus making parameter estimation difficult, or the estimates it gives for parameter vector are prone to give low prediction accuracy. Hence, regularization is a key process in solving such cases. It produces a sparse parameter vector and also shrinks the coefficients towards zero as well as towards each other [6].

### Regularized regression

The research on sparse and regularized solutions has gained increasing interest during the last ten years [7]. This is partly due to advances in measurement technologies, e.g., in molecular biology, where high-throughput technologies allow simultaneous measurement of tens of thousands of variables. However, the measurements are expensive, so typically the number of data points is small. In the field of bioprocess development, the number of variables is not that large but yet enough to hinder the use of many standard data analysis methods. Conventional regression and classification methods are unable to process data with more predictor variables than samples (so called *p *>>*N *problem). Regularization methods help in defining a unique solution in this ill-posed problem. These methods shrink some of the coefficients to zero. This not only helps in feature selection but also decreases the variance at the cost of a small increase in bias. However, this has the effect of improving the generalization of the estimate.

In regularized regression, a penalty on the size of the coefficients is added to the error function. Least absolute shrinkage and selection operator (LASSO) [3] is one such technique which uses the *L*_1 _norm of the coefficients as the penalty term to produce *sparse *solutions, i.e., prediction models with several coefficients equal to zero. Since variables with zero coefficients are not used, this procedure essentially acts as an embedded feature selection.

From the description of Equation (1), the *L*_1 _penalized coefficient vector for our linear model is defined as

(2) $$\hat{\theta }=\;||\;y-H\theta \;|{|}_{2}^{2}+\lambda ||\theta |{|}_{1}$$

where lambda (λ) is the regularization parameter, ||*θ *||_1 _is the *L*_1_-norm of the parameter vector. There exist efficient algorithms for finding solutions for different values of regularization parameters [3].

The result of the regularized regression is quite sensitive to the selection of the parameter λ. In order to appropriately assess the performance, the selection has to be done based on data. The usual approach is to estimate the performance with different λ using a cross-validation approach. Since we also use cross-validation for estimating the performance of the overall method (including the algorithm for selecting λ), this results in two nested cross-validation loops, one for model selection and one for error estimation. More specifically, the outer loop is used for estimating the performance for new data, while the inner loop is used for selection of λ.

### Random forests

Decision trees have been studied for decades as a model for various prediction problems. The tree can be either a classification tree or a regression tree, and a common term including both is classification and regression tree (CART). A decision tree is a hierarchical structure, which decides the class (in classification) or the predicted output (regression) by hierarchically comparing feature values with a selected threshold, thus producing a hierarchy of if-then rules. Such combination of rules is most conveniently expressed as a tree, where each input feature comparison corresponds to a node in the tree. Eventually, the leaves of the tree describe the actual output value.

The decision trees can be learned from the data, and the usual approach is to add nodes using a top-down greedy algorithm. In essence, this means dividing the search space into rectangular regions according to the splitting points. The drawback of decision tree is that they are very prone to overlearning. This is one reason why regression trees have later been extended to random forests [8], whose prediction is obtained by averaging the outputs of a large number of regression trees. Due to averaging, random forests are tolerant to overlearning, a typical phenomenon in high-dimensional settings with small sample size, and have thus gained popularity in classification and regression tasks especially in the area of bioinformatics.

In our experiments, we use the RF-ACE implementation in [5]. This implementation is very fast and it takes advantage of the Random Forest with Artificial Ensembles (RF-ACE) algorithm, which enables both feature ranking and model construction. In our approach, a set of significant features was first selected from the experimental data using the RF-ACE tool. Then, a model was constructed using the given data.

### Experimental data

In order to test our modeling methodology we examined a dataset produced in a study related to culture media optimization (unpublished data, Rahul Mangayil et al.). There, an enriched mixed microbial consortium was used in the bioconversion of crude glycerol to hydrogen, and the process was optimized in serum bottles by optimization of media components. The concentrations of five media components (NH_4_Cl, K_2_HPO_4_, KH_2_PO_4_, MgCl_2_.6H_2_O, and KCl) were varied with the help of statistical design of experiments (Plackett-Burman, steepest ascent, Box-Behnken), and the resulting hydrogen production was measured (in mol-H_2_/mol-glycerol). The data was modeled using first and second order polynomials in multiple linear regression. This data containing 35 samples is a typical data set produced during bioprocess modeling and optimization. Multiple linear regression is a useful tool for modeling the data from individual designs of the study but other methods are needed in order to model the entire data set at once.

### Visualization and validation of models

In order to provide an overview to the models and the experimental data, visual representations were produced for the regularized regression model and the random forest model. Since visualization of the high dimensional variable space (five dimensions in our case study) is not feasible, the variables are visualized pair-wise. The values of remaining variables (three) are set in their average values calculated from the data. In addition, each model is assessed with *leave-one-out *(LOO) cross validation technique which estimates the accuracy of the predictions in an independent dataset.

## Results and discussion

In our case study, we used multiple linear regression, regularized regression and random forests to predict the yield of hydrogen production. The performance of each method is evaluated by original dataset as well as transformed dataset with pairwise interactions and quadratic forms. Therefore, the original dataset contains 5 variables while the transformed dataset contains 20 variables.

### Yield prediction using multiple linear regression

Multiple linear regression is used with and without non-linearly transformed predictor variables to model the response variable. Without the transformed predictors, i.e., the simple model, the estimated correlation value (using the LOO cross-validation) was 0.65. However, using the transformed polynomial model the estimate for correlation decreased to a very low value of 0.012 and resulted in an insignificant model. This is mainly due to the aforementioned shortcomings of the multiple linear regression. It basically over-fits the model to the training samples and thus produces less accurate estimates for unseen data samples. Table S1 lists the model coefficients for the transformed polynomial regression model [see Additional file 1]. It can be noted that zero entries have been inserted to remove linearly dependent observations.

### Yield prediction using regularized regression

First, we evaluated the simple model without the transformed variables. In this case, the parameter λ for the regularized regression is chosen by both manual selection and proper cross validation. In other words, we wanted to see if the results improve by manually selecting the lambda value optimally for each LOO cross validation fold. Although this is not possible in practical applications, it may give insight on the efficiency of parameter selection using cross-validation with small sample size, and on the general applicability of a linear model for our problem.

As a result, the LOO correlation estimate becomes 0.85 with manual selection instead of 0.60 using proper cross-validation. The large gap between optimal and estimated correlation is at least in part due to the inaccuracy of the cross-validation type error estimators with small sample size; see, e.g., [9].

**Figure 1:**
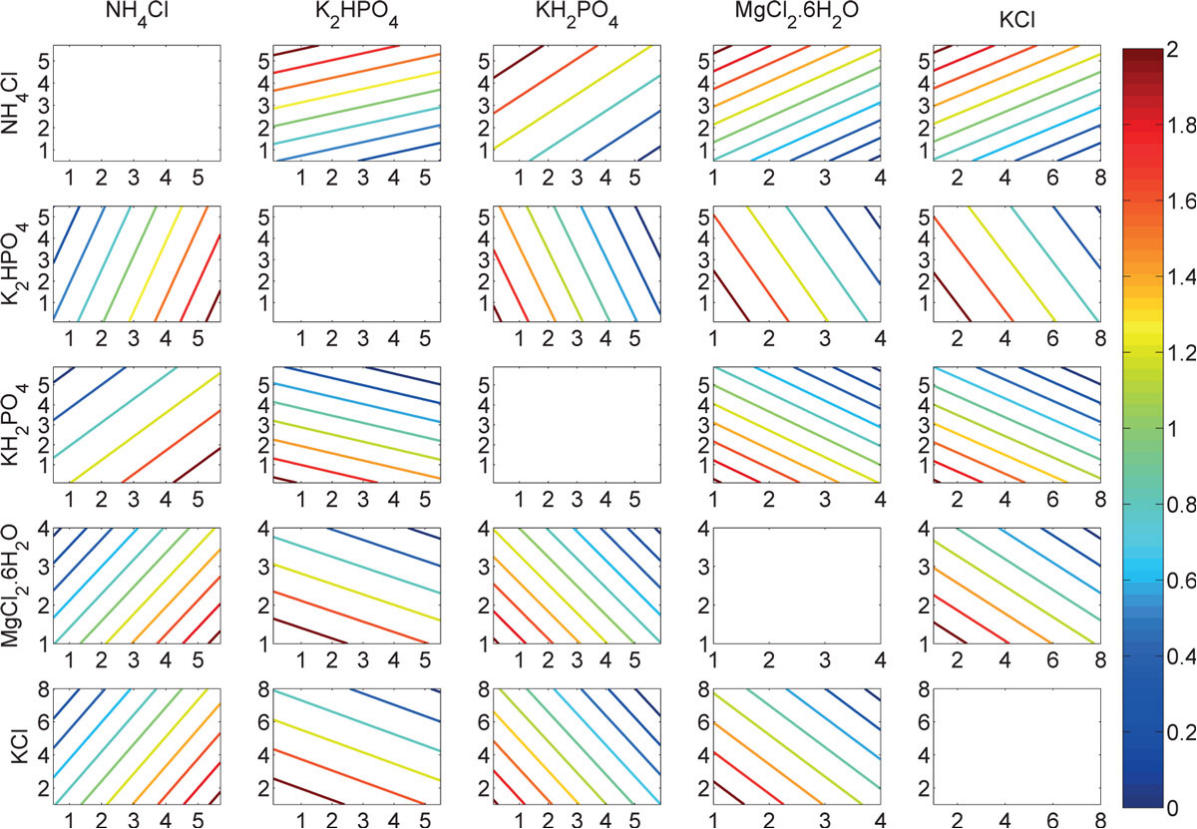
**Yield predictions using the regularized regression model**. The yields are presented by different colors according to the colorbar. The plots in the diagonal (i.e., variables are plotted against themselves) are left empty.

In the case of transformed polynomial regression model, the estimated value for correlation was found to be 0.69 which is higher than the case of the simple model. This clearly indicates the non-linear behavior of the original dataset. Table S1 shows the resulting coefficients in the constructed model where regularization has forced 5 out of 21 coefficients to zero [see Additional file 1]. Although, the same number of non-zero coefficients were obtained from the multiple linear regression as well but the main difference is the regularized coefficients. That is, the non-zero coefficients from regularized regression were also shrunk towards zero. This results in generalized models with higher overall prediction accuracy [3]. The yield predictions are visualized in Figure 1 as a response surface. In addition, the significant variables for the model and their corresponding coefficients are listed in Table 1.

**Table 1:** Significant variables and their coefficients in the regularized regression model

Significant variables	Coefficient values
NH_4_Cl	0.1254
K_2_HPO_4_	-0.0383
KH_2_PO_4_	-0.1061
MgCl_2_.6H_2_O	-0.1418
KCl	-0.0562

### Yield prediction using random forests

**Figure 2:**
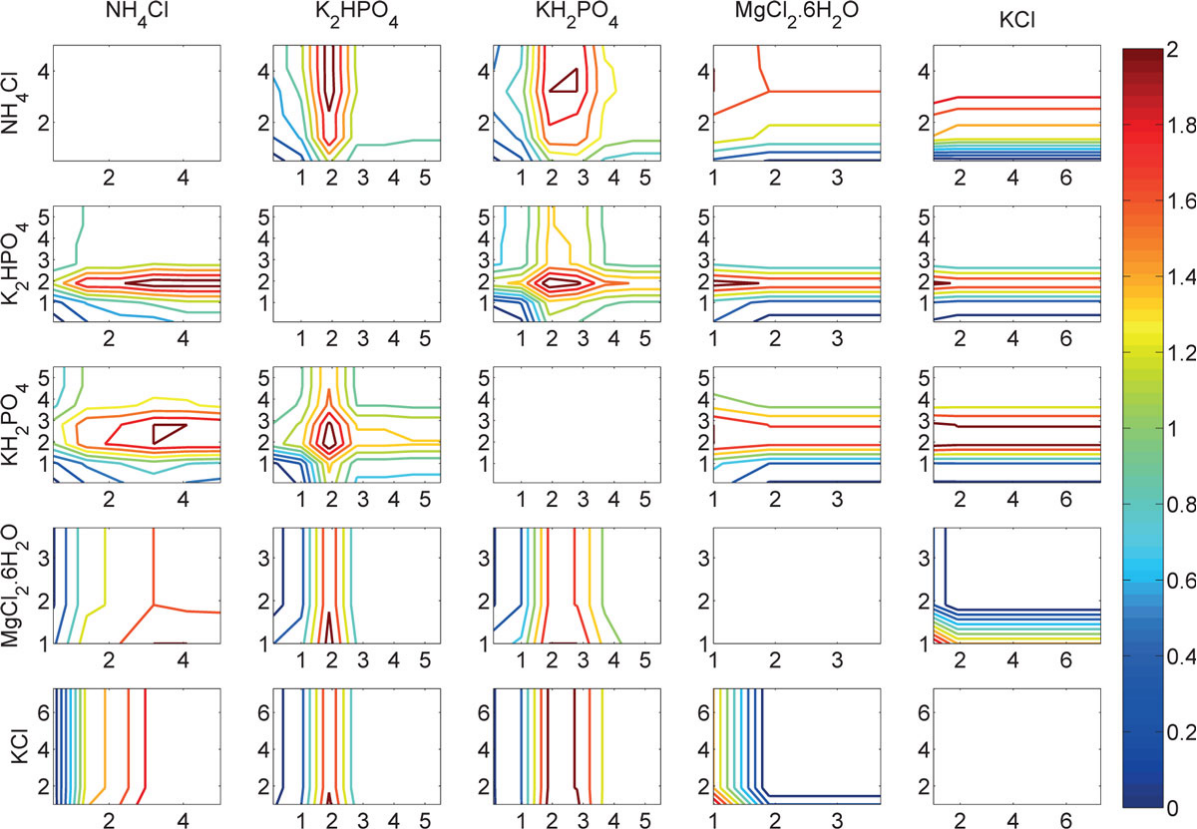
**Yield predictions using the random forest model**. The yields are presented by different colors according to the colorbar. The plots in the diagonal (i.e., variables are plotted against themselves) are left empty.

The RF-ACE tool [5] is used to build the random forests model. In our experiment, the type of the forest, the number of trees in the forest, and the fraction of randomly drawn features per node split are set to "RF", 20, and 10, respectively. All other parameters were kept to their default values. The results indicated that all variables were significant in the model. The yield predictions of the constructed model are visualized in Figure 2. In the accuracy examination, the RF-ACE model resulted in correlation of 0.88 (using LOO cross-validation). The capability of modeling non-linear relationships is the primary reason for high prediction accuracy in the constructed model. On the other hand, the model provided correlation value of 0.91 if the variable transformations were used as additional predictor variables. Eventually, the increase is quite small, and may thus be a due to random fluctuation.

### Method comparison

**Figure 3:**
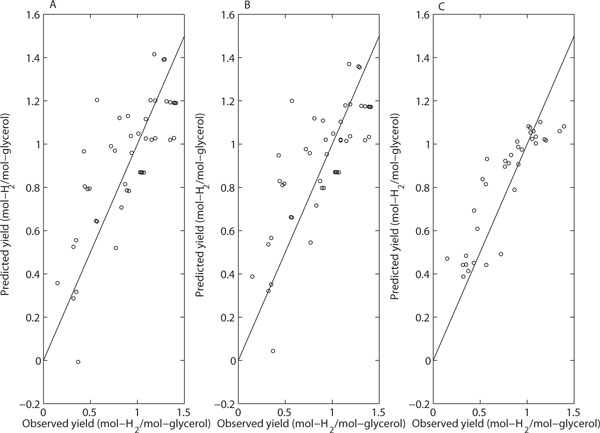
**Comparison of prediction performance of models obtained by three methods for original dataset**. **(A) **Multiple Linear Regression; **(B) **Lasso; **(C) **Random Forest. The straight line depicts perfect predictions should lie. The prediction accuracy for each model is estimated using LOO cross-validation.

**Figure 4:**
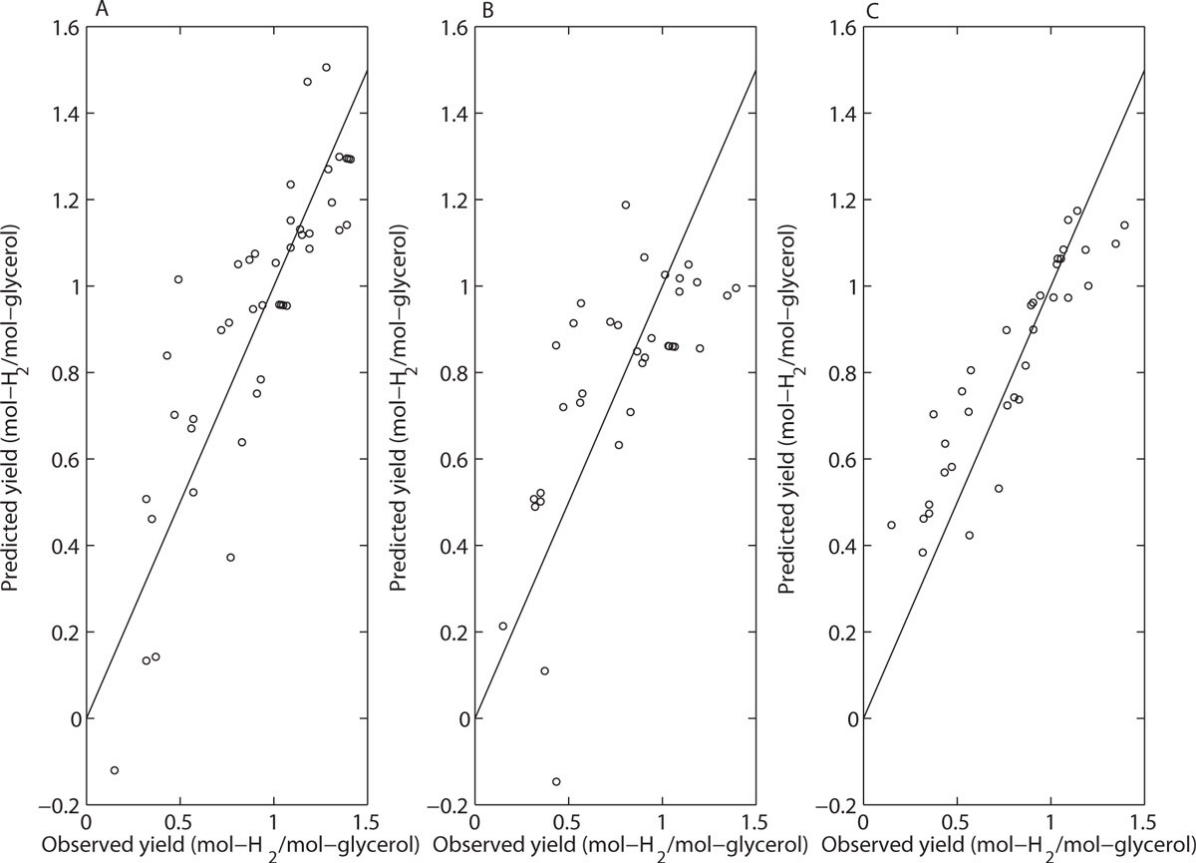
**Comparison of prediction performance of models for the dataset containing the actual and the transformed variables**. **(A) **Multiple Linear Regression; **(B) **Lasso; **(C) **Random Forest. The straight line depicts perfect predictions should lie. The prediction accuracy for each model is estimated using LOO cross-validation.

Both regularized regression with transformed variables and random forests produced results that are useful in bioprocess data mining. In particular, both methods determined all the variables significant and can be used to determine an advantageous control direction for them. The most notable difference in the results is the linearity that was in use in the regularized regression versus the nonlinearity that is inherent in random forests (see Figures 3 and 4). Simple linear models cannot fit to the nonlinearity of the data and, thus, the maximum response cannot be detected inside the examined space although it would be located in there. However, regularized linear regression with transformed variables was found successful in modeling the nonlinearity of the data to some extent. On the other hand, the random forest model is able to capture the nonlinearity. Here, the maximum response was determined approximately at the same point as in the media optimization study performed using the methods of statistical design of experiments.

Figure 3 and 4 show the performance of the three methods in yield prediction. It is clear that regularized linear regression failed to cope with data non-linearity unless transformed variables were used in regression. On the other hand, the use of transformed variables causes the multiple linear regression to fail. Thus, multiple linear regression is an efficient tool in the analysis of individual datasets designed by statistical design of experiments (e.g., Plackett-Burman and Box-Behnken) but not useful in data mining of more complicated datasets like the one examined in here.

The LOO estimates for correlation ascertain that the RF-ACE provides a more accurate solution than the regularized regression. This, however, should not totally renounce the idea of using regularized regression as it mainly proves its worth in more complicated and high-dimensional data analysis. Moreover, linear regression has a useful feature of producing easily interpretable models and, on the other hand, the models are capable in producing predictions beyond the already examined parameter space.

## Conclusions

In this study, we applied two novel data analysis methods (regularized regression and random forests) in bioprocess data mining and compared them to multiple linear regression that is commonly applied in relation to statistical design of experiments. Both of the studied methods were able to produce models that fit to the examined data. In particular, the non-linearity of the data was well modeled by random forests. This property is very valuable in data mining of multiple integrated data sets. As the results demonstrated, traditionally used multiple linear regression does not perform satisfactorily in nonlinear input-output relations. The traditional approach using the first and the second order polynomial models would face further problems if the data was multimodal. In the future, it would be of interest to further study regularized regression and random forests in bioprocess data mining. This could mean, for example, the inclusion of categorical variables in the data and studies with different types of bioprocesses.

## Competing interests

The authors declare that they have no competing interests.

## Authors' contributions

SSH and MF made substantial contribution in writing the manuscript, interpretation of the data, and design and analysis of the models. RM was responsible in acquisition of the data. TA and HH contributed to the design of the study, and in writing and revising the manuscript.

## Supplementary Material

Additional file 1 **as PDF - Table S1: Significant coefficient values in different methods using transformed data**. This file contains a table describing the coefficient values generated by Lasso and multiple linear regression methods for the transformed dataset. Here, the coefficient *β_0 _*represents the intercept, *β_1 _*corresponds to variable NH_4_Cl, *β_2 _*to K_2_HPO_4_, *β_3 _*to KH_2_PO_4_, *β_4 _*to MgCl_2_.6H_2_O and *β_5 _*to KCl, respectively. Click here for file

## Declarations

The publication cost for this work was supported by the Academy of Finland, project "Butanol from Sustainable Sources" (decision number 140018).

This article has been published as part of BMC Systems Biology Volume 7 Supplement 1, 2013: Selected articles from the 10th International Workshop on Computational Systems Biology (WCSB) 2013: Systems Biology. The full contents of the supplement are available online at http://www.biomedcentral.com/bmcsystbiol/supplements/7/S1.
