# Supplementary material for: Bioprocess data mining using regularized regression and random forests
Source: BMC Syst Biol. 2013 Aug 12;7(Suppl 1):S5. doi: 10.1186/1752-0509-7-S1-S5 (PMC3750505; doi:10.1186/1752-0509-7-S1-S5)
Supplement: Additional file 1 — as PDF - Table S1: Significant coefficient values in different methods using transformed data. This file contains a table describing the coefficient values generated by Lasso and multiple linear regression methods for the transformed dataset. Here, the coefficient β0 represents the intercept, β1 corresponds to variable NH4Cl, β2 to K2HPO4, β3 to KH2PO4, β4 to MgCl2.6H2O and β5 to KCl, respectively. [file 1752-0509-7-S1-S5-S1.PDF]

Supplementary data

Table S1: Significant coefficient values in different methods using transformed data. This file contains a table describing the coefficient values generated by Lasso and multiple linear regression methods for the transformed dataset. Here, the coefficient β_0 represents the intercept, β_1 corresponds to variable NH_4Cl, β_2 to K_2HPO_4, β_3 to KH_2PO_4, β_4 to $MgCl_2.6H_2O$ and β_5 to KCl, respectively.

Coefficient terms	Lasso	Multiple linear regression
β_0	0.5896	0.5491
β_1	0	-0.0188
β_2	0.1594	0.8297
β_3	0.1542	-0.5572
β_4	-0.0377	0
β_5	-0.0343	0
β_1^2	-0.0044	-0.0030
β_2^2	-0.1077	-0.4467
β_3^2	-0.0635	-0.0694
β_4^2	-0.0129	-0.0380
β_5^2	-0.0001	0.0052
$\beta_1 \beta_2$	0.0947	0.1057
$\beta_1 \beta_3$	0	0.004
$\beta_1 \beta_4$	0.0073	0
$\beta_1 \beta_5$	0.0068	0
$\beta_2 \beta_3$	0.0259	0.3367
$\beta_2 \beta_4$	0	0
$\beta_2 \beta_5$	-0.0055	-0.1103
$\beta_3 \beta_4$	0	0.2323
$\beta_3 \beta_5$	0.0049	-0.0116
$\beta_4 \beta_5$	0	-0.0037
